# Personalized Predictions for Changes in Knee Pain Among Patients With Osteoarthritis Participating in Supervised Exercise and Education: Prognostic Model Study

**DOI:** 10.2196/60162

**Published:** 2025-03-21

**Authors:** Mahdie Rafiei, Supratim Das, Mohammad Bakhtiari, Ewa Maria Roos, Søren T Skou, Dorte T Grønne, Jan Baumbach, Linda Baumbach

**Affiliations:** 1 Faculty of Mathematics, Informatics and Natural Sciences, Institute for Computational Systems Biology, University of Hamburg, Albert-Einstein-Ring 8-10, Hamburg, 22761, Germany, 49 40428387370; 2 Department of Health Economics and Health Services Research, University Medical Center Hamburg-Eppendorf, Hamburg, Germany; 3 Center for Muscle and Joint Health, Department of Sports Science and Clinical Biomechanics, University of Southern Denmark, Odense, Denmark; 4 The Research and Implementation Unit PROgrez, Department of Physiotherapy and Occupational Therapy, Næstved-Slagelse-Ringsted Hospitals, Slagelse, Denmark; 5 Computational Biomedicine Lab, Department of Mathematics and Computer Science, University of Southern Denmark, Odense, Denmark; 6 Chair of Genome Informatics, Center for Bioinformatics, University of Hamburg, Hamburg, Germany

**Keywords:** osteoarthritis, prediction, pain intensity, exercise therapy, machine learning

## Abstract

**Background:**

Knee osteoarthritis (OA) is a common chronic condition that impairs mobility and diminishes quality of life. Despite the proven benefits of exercise therapy and patient education in managing OA pain and functional limitations, these strategies are often underused. To motivate and enhance patient engagement, personalized outcome prediction models can be used. However, the accuracy of existing models in predicting changes in knee pain outcomes remains insufficiently examined.

**Objective:**

This study aims to validate existing models and introduce a concise personalized model predicting changes in knee pain from before to after participating in a supervised patient education and exercise therapy program (GLA:D) among patients with knee OA.

**Methods:**

Our prediction models leverage self-reported patient information and functional measures. To refine the number of variables, we evaluated the variable importance and applied clinical reasoning. We trained random forest regression models and compared the rate of true predictions of our models with those using average values. In supplementary analyses, we additionally considered recently added variables to the GLA:D registry.

**Results:**

We evaluated the performance of a full, continuous, and concise model including all 34 variables, all 11 continuous variables, and the 6 most predictive variables, respectively. All three models performed similarly and were comparable to the existing model, with *R*^2^ values of 0.31‐0.32 and root-mean-squared errors of 18.65‐18.85—despite our increased sample size. Allowing a deviation of 15 (visual analog scale) points from the true change in pain, our concise model correctly estimated the change in pain in 58% of cases, while using average values that resulted in 51% accuracy. Our supplementary analysis led to similar outcomes.

**Conclusions:**

Our concise personalized prediction model provides more often accurate predictions for changes in knee pain after the GLA:D program than using average pain improvement values. Neither the increase in sample size nor the inclusion of additional variables improved previous models. Based on current knowledge and available data, no better predictions are possible. Guidance is needed on when a model’s performance is good enough for clinical practice use.

## Introduction

### Background

Osteoarthritis (OA) is a chronic disease characterized by persistent joint pain and limited mobility, especially for those who experience from OA of the knee [1]. In the United States alone, the overall economic burden of OA is estimated at almost US $140 billion annually, highlighting the substantial societal and personal costs associated with the condition [2]. Pain is the most important outcome for patients with OA, with moderate to severe pain associated with greater functional limitations, treatment dissatisfaction, and reduced quality of life [3]. In managing OA, patient education and exercise therapy, which are the recommended first-line treatments according to clinical guidelines, play a critical role [4-6]. Treatment programs that combine therapeutic exercise with other treatments such as weight loss [7], manual therapy, and pain education have shown promising results in managing mild to moderate OA symptoms and provide complementary options to improve patient’s pain, function, and quality of life [8,9]. Patient education further empowers individuals by enhancing their understanding of the disease and emphasizing the importance of lifestyle adjustments such as a balanced diet and regular exercise [10].

### Prior Work

However, the implementation of recommended treatments could be improved [11]. The discrepancy between recommended and provided treatments has been attributed to several factors, including health care providers offering no evidence-based care and patients’ lack of motivation and awareness about the long-term benefits of the recommended therapies [12,13]. Furthermore, some patients are hesitant to participate in exercise therapy programs, either because they are uninterested or face logistical difficulties [14,15]. It is relevant to develop strategies that assist clinicians and patients in an engaging way in the treatment decision process such as offering personalized outcome predictions.

Digital technology presents a promising solution to address such challenges in health care. Prediction models, particularly, have emerged as powerful tools to support patients and health care professionals in the treatment decisions and management of chronic diseases like OA [16-18].

### The Goal of This Work

The GLA:D (Good Life with osteoArthritis in Denmark) program has garnered significant attention as a pioneering initiative in OA management [19]. Comprising three components—training for physiotherapists, a patient education and neuromuscular exercise therapy program delivered to patients, and clinical data collection—the program stands out as an example of evidence-based care [19]. To support shared decision-making, the acceptance, and participation rate of programs like GLA:D, we aimed to validate an existing model and introduce an updated concise personalized prediction model that estimates changes in knee pain intensity for patients with OA considering participation in the GLA:D program [20].

## Methods

### Overview

This paper follows the guidelines of TRIPOD (Transparent Reporting of a Multivariable Prediction Model for Individual Prognosis or Diagnosis) [21].

In this paper, we reproduced and validated the results of previous work [20] with some methodological changes as described below.

### Source of Data and Participants

We used data from the Danish GLA:D initiative for patients with knee and hip OA. The initiative consists of a 2-day course for physiotherapists, a patient treatment program delivered in clinical practice, and a registry of data reported by patients and clinicians. The patient program combines 2 patient education and 12 supervised exercise therapy sessions aiming at improving symptoms, physical function, and quality of life. Patients can join the GLA:D program through referrals from health care professionals (eg, general practitioners or orthopedic surgeons) or via self-referral, followed by an assessment by a certified GLA:D clinician to confirm the OA diagnosis [22-25]. By 2023, in total, 257 clinics were offering the GLA:D program for knee or hip OA [26]. Of these, most clinics were private practices, but 20 clinics were public municipalities, and 2 municipalities offered the treatment for employees.

The GLA:D registry collects data from participants, which includes demographic details, medical history, pain intensity, and physical function measures [27]. The data are collected at three time points: at baseline, immediately following the program, and at 12 months follow-up. GLA:D was established in 2013 and is regularly updated to include new evidence. Comprehensive details about GLA:D’s education, neuromuscular exercise program, and general information are previously available [19]. For this paper, we followed the inclusion criteria of a previous publication [20] but with an extended inclusion period from October 9, 2014, to November 12, 2022, instead of ending on August 31, 2017. We included patients who indicated their knee as the primary joint of complaint and provided complete data. We excluded participants for specific time periods due to technical problems in the registry, meaning one of our variables of interest was not collected. This was the case between May 13, 2016, and November 12, 2016, and April 10, 2018, and August 05, 2019.

### Variable Selection

#### Outcome

“Change in pain intensity” was our outcome of interest. This measure was calculated by determining the difference in pain scores on the visual analog scale (VAS) from baseline to immediately after the program (after about 3 mo). The question asked to the patients was “On a scale that goes from no pain (0 mm) to worst pain imaginable (100 mm), what score best represents your knee pain during the last week?” [28]. We examined various thresholds, ranging from 5 to 20 mm, to define a significant change in pain intensity.

#### Predictor Variable Selection

Our analysis incorporated 34 potential predictor variables: 11 continuous, 22 binary, and 1 with three categories. These variables were chosen for their relevance to knee OA and health outcomes, encompassing factors like clinical symptoms, lifestyle influences, and demographic details, to provide a comprehensive understanding of the disease’s impact and patient outcomes. In comparison to the earlier study, we included 17 fewer variables (diabetes, S12 physical component and mental score, American Shoulder and Elbow Surgeons Shoulder Score pain and other symptoms score, and some medical disease) since their collection stopped (for 4 variables in 2018, for 2 variables in 2020, and for 12 variables, their collection stopped in 2021).

#### Selection of Important Variables

We used random forest regressions during variable selection and model development. The random forest regressor is an ensemble model that efficiently reduces variables without overfitting and captures complex, nonlinear relationships between predictors and the outcome variable [29]. Random forest regression was previously used on the GLA:D dataset to predict changes in VAS pain considering 51 variables in the training process; its performance is similar to a linear regression [20]. The preprocessing of our dataset was performed according to the previous study results, which we aim to validate [20].

We applied a 2-step variable selection process to optimize our predictive model and attempt to achieve clinical acceptance. Initially, we started with 34 predictor variables. To reduce the number of variables, we first evaluated the variable importance using Gini impurity [30]. The Gini index or impurity measures the probability of a random instance being misclassified when chosen randomly. The Gini impurity method evaluates the importance of each variable by examining how much it contributes to reducing impurity when used as a split criterion in decision trees [31]. Variables with higher Gini importance scores are considered more important in making accurate predictions. In many implementations, like scikit-learn’s random forest, the Gini importance scores are normalized such that they all sum up to 1. Therefore, each variable’s importance score will range between 0 and 1, with higher values indicating greater importance. We built and trained random forest regressor models, incorporating the top k variables, with k determined using the elbow method. The elbow method is a straightforward technique for determining the optimal number of variables in a model. It involves identifying the point at which adding more variables leads to diminishing returns, meaning their contribution to overall model performance becomes negligible [32]. To enhance the robustness of our analysis, we used cross-validation during the variable importance assessment.

The Gini coefficient is mathematically represented as:


$$Gini=1-\sum_{i=1}^{C}{\left(p_{i}\right)}^{2}$$

In the second step, we respected the importance of the individual variables and the elbow technique, as well as applying clinical reasoning to reduce and find the ideal set of variables. While this reduction of variables could potentially reduce the model’s performance, we limited the number of questions, such that it would be convenient for end users to use the model, thereby increasing the likelihood of its clinical value.

With this 2-step approach, incorporating both Gini impurity and clinical reasoning, we aimed to achieve a balanced model that is both predictive and user-friendly.

### Model Development and Evaluation

To develop and evaluate our predictive model, we began by splitting our dataset into two subsets: a training set for model development and a test set for validation. The training process was consistently applied on all models, regardless of training on all variables, on the top k predictor variables, or on a concise set of variables. To enhance the robustness and generalizability of our models, we integrated 10-fold cross-validation with random forest regression. Using the test sets, we validated our findings, ensuring that our results were not biased or limited to specific data instances by averaging results across the cross-validation test sets.

To evaluate the performance of the models, we used the root-mean-squared error (RMSE) and *R*^2^ metrics. RMSE measures the average deviation between the predicted and actual values, providing an assessment of the model’s accuracy. The *R*^2^ represents the proportion of variance the model explains and indicates how well the model fits the data. To compare our personalized outcome predictions with predictions of the average model, we evaluated the number of correct predictions, in contrast to the previous publication, which used the absolute mean differences in a test dataset [20].

The predicted changes in pain intensity by our random forest regression constitute the personalized predictions. The average model prediction was defined as a mean function of the VAS pain change score of the training samples, $\mu =\frac{\sum_{n=1}^{N}VASChangeScore}{N}$. The average model always provides *μ*, regardless of the features of the unseen samples, as the predicted change in pain intensity. In this paper, we are always focused on the VAS pain change score, rather than the pain score at a specific time point. Recognizing that predictions inherently have some margin of error, we incorporated in our evaluation a margin of error that corresponds to a clinically relevant threshold.


$$I_{n}=\left\{ \begin{array}{ll} 1: & \text{if }L_{n}\leq {Pred}_{n}\leq U_{n}\\ 0: & \text{otherwise} \end{array}\right.$$

$I_{n}$ is the indicator value that will be one for cases where their predicted change in pain value is inside the interval. For all other samples, it is zero. $L_{n}=VASChangeScore-\;R$ and $U_{n}=VASChangeScore\;+\;R$ show the lower and upper bound based on the clinical relevance threshold *R*. Finally, we calculate the percentage of predictions inside the interval ρ to evaluate and compare our model against the average model:


$$\rho =\frac{\sum_{n=1}^{N}I_{n}}{N}.$$

Since the value of a clinical relevance *R* is debatable and ranges up to 20 mm [33-35] points, we evaluated the number of correct predictions with different tolerable margins of error, ranging from 5 to 20 points. Thus, we calculated the number of correct predictions for several cases, the first allowing a deviation of 5 points and the last allowing a deviation of 20 points from the true change in pain.

All data analyses were performed in Python (Python Software Foundation) using Scikit-learn, Pandas, and NumPy libraries [36]. We used Pandas and NumPy for data preprocessing tasks, such as cleaning and variable scaling. Scikit-learn was used for validation techniques, including train-test split, cross-validation, and stratified sampling. We used standard hyperparameters for the random forest regression: n_estimators set to 100, max_depth limited to 10, and random_state fixed at 42. We chose to use them to provide a baseline performance that can be compared with other standard implementations.

### Supplementary Analyses

To evaluate if the inclusion of recently added variables to the GLA:D registry improved our outcome predictions, we additionally developed and evaluated models following the same process as described above, but including 12 additional variables (load-related pain, reduced functional capacity, morning stiffness, crepitus, reduced knee movement, bony enlargement, previous joint injury, occupational or recreational overuse, family members with OA, Knee Injury and Osteoarthritis Outcome Score-12 pain, functioning, and Knee Injury and Osteoarthritis Outcome Score-12 summary score, which were added to the registry in May 2018). A flowchart for this additional analysis is provided in Figure S2 in Multimedia Appendix 1 [37].

### Ethical Considerations

This study did not require ethics approval or clinical trial registration, as determined by the local ethics committee of the North Denmark Region, since it was a register-based study rather than a clinical trial. The GLA:D registry has been approved by the Danish Data Protection Agency, ensuring compliance with data protection regulations. In accordance with the Danish Data Protection Act, patient consent was not required, as personal data were processed solely for research and statistical purposes. To protect participant confidentiality, all data were anonymized before analysis, with no access to personally identifiable information. Data handling followed the regulations of the Danish Data Protection Agency. No compensation was provided to participants, as this study relied on secondary data analysis of the GLA:D registry.

## Results

**Figure 1.**
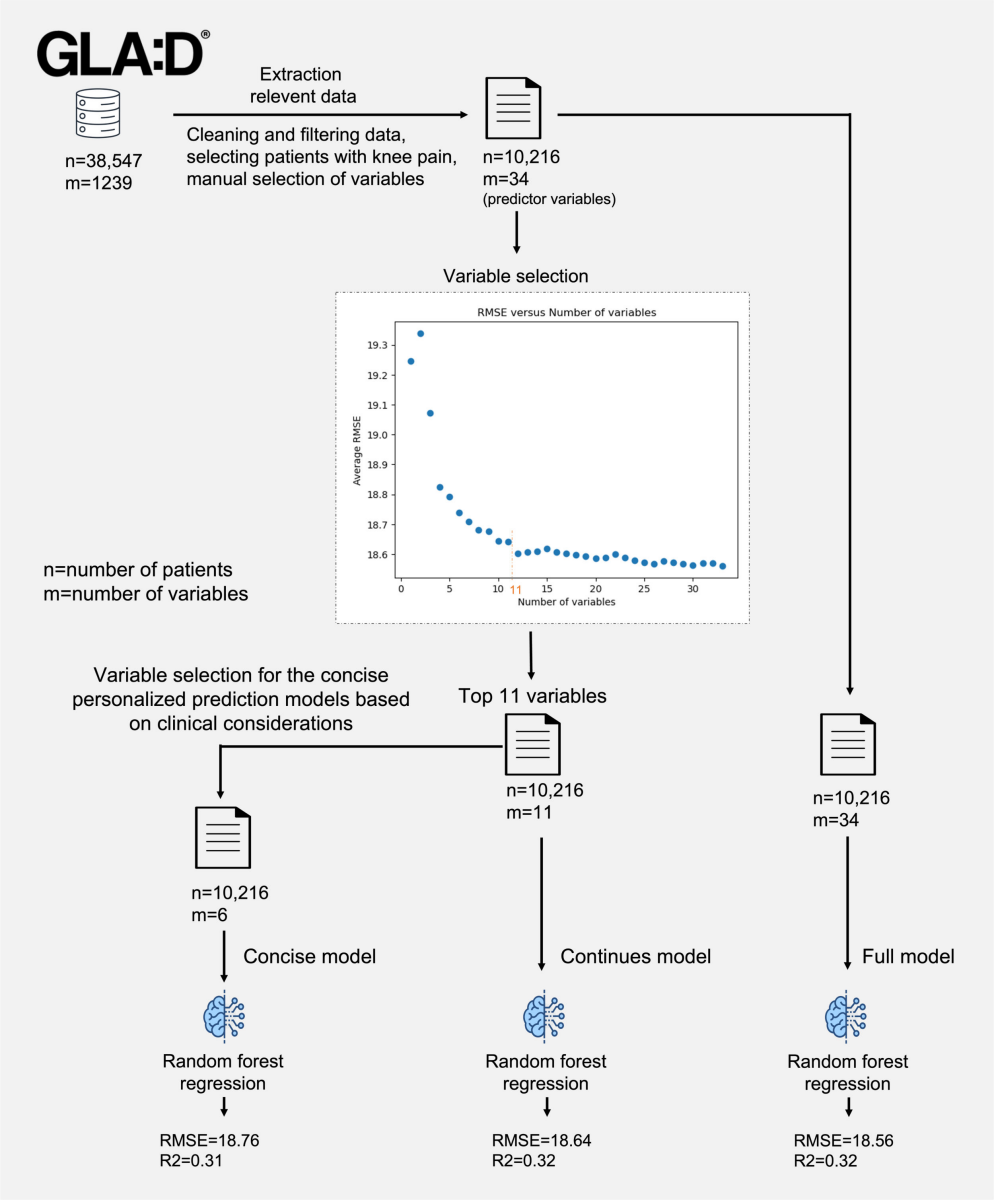
A brief overview of the personalized prediction to predict changes in knee pain in patients with knee osteoarthritis. RMSE: root-mean-squared error.

A brief overview of our results is displayed in Figure 1.

### Source of Data and Participants

**Figure 2.**
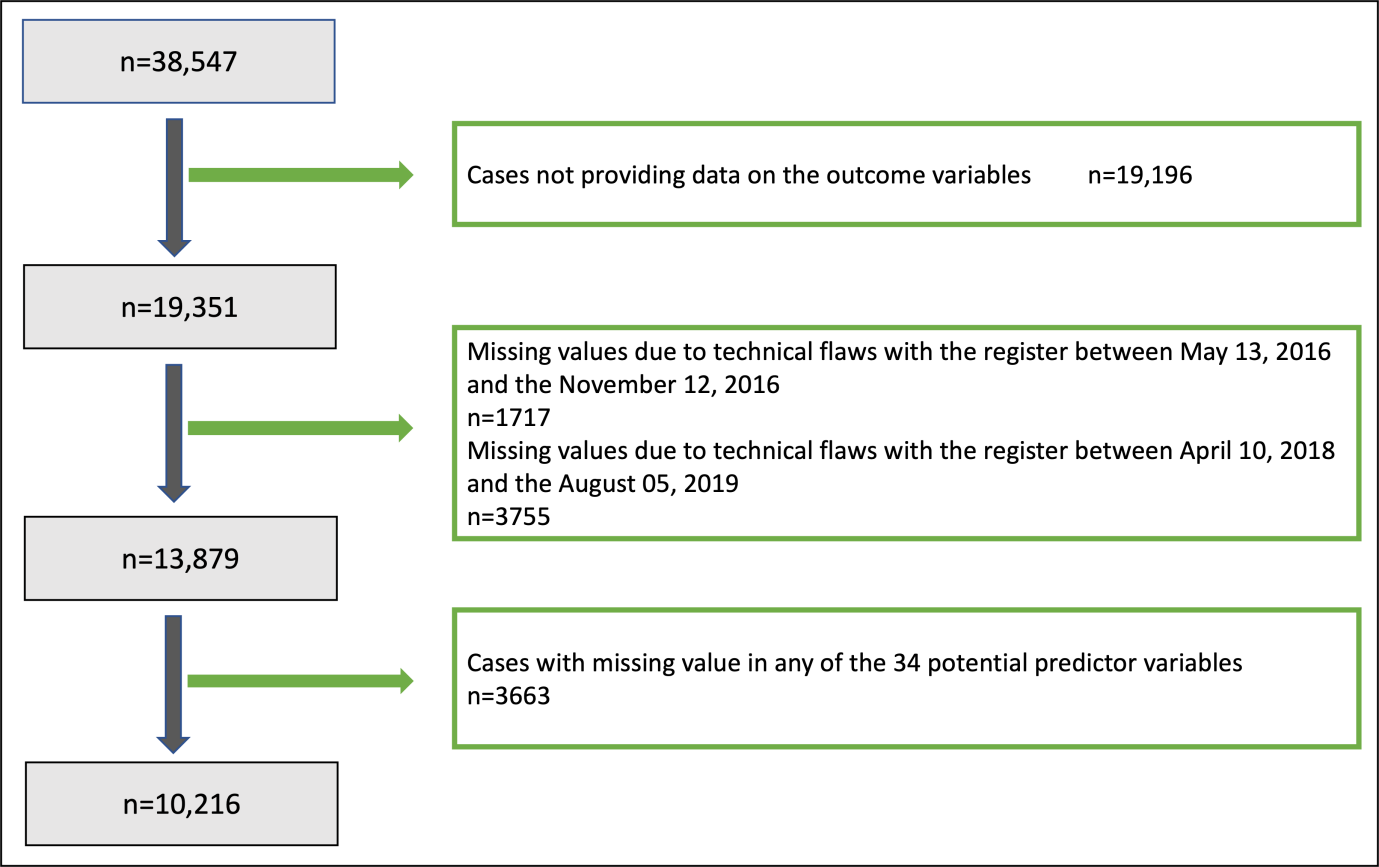
Flowchart of patient selection and data exclusion criteria for the study, showing initial cohort size and subsequent exclusions due to specific criteria and missing data.

A total of 38,547 patients fulfilled our inclusion criteria for participating in the GLA:D program between October 9, 2014, and November 12, 2022. We considered patients with knee pain as the primary complaint and our analytical dataset was reduced to 10,216 patients. Details on the process of patient inclusion and exclusion are available in Figure 2.

### Variable Selection

**Figure 3.**
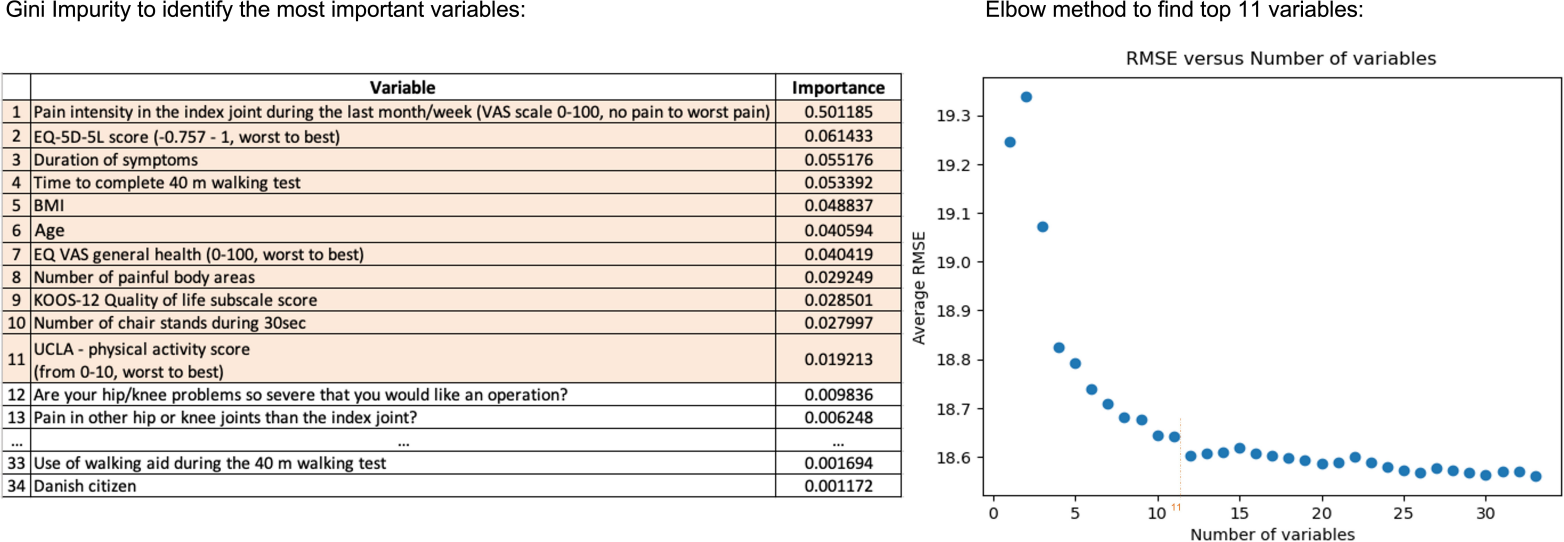
Demonstrates the 2-fold analytical process where the left panel presents the ranking of variables by their relative importance using Gini impurity. Following this initial analysis, the elbow method, showcased in the right panel, was applied. KOOS: Knee Injury and Osteoarthritis Outcome Score; RMSE: root-mean-squared error; UCLA: University of California, Los Angeles; VAS: visual analog scale.

The Gini impurity scores for all variables are presented in Figure S2 in Multimedia Appendix 1 [37]. The characteristics of all included and excluded samples are provided in Table 1. Based on these findings, we applied the elbow method and included the top 11 variables (Figure 3).

**Table 1.** Characteristics of all included and excluded participants.

Variable	Included cases (n=10,216)	Excluded cases (n=28,331)
Predictor variables		
Age		
Mean (SD)	65.01 (9.36)	65.08 (9.81)
Range (min-max)	23‐94	18‐100
Missing, n (%)	—^a^	0 (0)
BMI		
Mean (SD)	28.61 (5.28)	29.03 (5.53)
Range (min-max)	15.23‐70.03	14.41‐72.27
Missing, n (%)	—	218 (0.77)
Sex, n (%)		
Male	2892 (28.31)	8606 (30.38)
Female	7324 (71.69)	19,725 (69.62)
Missing	—	0 (0)
Duration of symptoms: how long has the patient had the symptom in the most painful joint? (months)		
Mean (SD)	43.05 (66.91)	34.34 (56.65)
Range (min-max)	0.0‐756.0	0.0‐840.0
Missing, n (%)	—	3170 (11.19)
Waitlisted for knee or hip surgery, n (%)		
Yes	174 (1.70)	353 (1.25)
No	10,042 (98.30)	19,365 (68.35)
Missing	—	8613 (30.40)
Radiographic signs of knee OA^b^, n (%)		
Yes	8020 (78.50)	22,161 (78.22)
No	396 (3.88)	925 (3.26)
Unknown	1800 (17.62)	4947 (17.46)
Missing	—	298 (1.05)
Previous contact with physiotherapist because of the current joint problems, n (%)		
Yes	3437 (33.64)	9118 (32.18)
No	6779 (66.36)	19,212 (67.81)
Missing	—	1 (0.003)
Use of painkillers (paracetamol or NSAID^c^ or opioids) in the last three months, n (%)		
Yes	6382 (62.47)	17,638 (62.26)
No	3834 (37.53)	10,693 (37.74)
Missing	—	0 (0)
Prior surgery in the index joint, n (%)		
Yes	2842 (27.82)	7390 (26.08)
No	7374 (72.18)	20,941 (73.92)
Missing	—	0 (0)
Time to complete 40-m walking test		
Mean (SD)	28.23 (7.98)	29.04 (8.80)
Range (min-max)	10.0‐234.91	10.0‐221.02
Missing	—	2452 (8.65)
Use of walking aid during the 40-m walking test, n (%)		
Yes	177 (1.73)	534 (1.88)
No	10,039 (98.27)	26,009 (91.80)
Missing	—	1788 (6.31)
Number of chair stands during 30 seconds		
Mean (SD)	11.97 (3.70)	11.81 (3.95)
Range (min-max)	0.0‐35.0	0.0‐40.0
Missing	—	1794 (6.33)
Born in Denmark, n (%)		
Yes	9844 (96.36)	27,102 (95.66)
No	372 (3.64)	1221 (4.31)
Missing	—	8 (0.03)
Danish citizen, n (%)		
Yes	10,055 (98.42)	27,827 (98.22)
No	161 (1.58)	496 (1.75)
Missing	—	8 (0.03)
Living situation (are you living alone or with others?)^d^, n (%)		
Yes	2461 (24.09)	7409 (26.15)
No	7755 (75.91)	20,914 (73.82)
Missing	—	8 (0.03)
Educational level: Do you have a higher education than secondary education? n (%)		
Yes	7266 (71.12)	13,907 (49.09)
No	2950 (28.88)	5802 (20.48)
Missing	—	8622 (30.43)
Smoking, n (%)		
Yes	787 (7.70)	2567 (9.06)
No	9429 (92.30)	25,757 (90.91)
Missing	—	7 (0.02)
Previous injury in the index joint that caused you to consult a medical doctor, n (%)		
Yes	5268 (51.57)	14,500 (51.18)
No	4948 (48.43)	13,818 (48.77)
Missing	—	13 (0.05)
Pain in hip or knee other than the index joint?, n (%)		
Yes	5906 (57.81)	16,478 (58.16)
No	4310 (42.19)	11,853 (41.84)
Missing	—	0 (0)
Walking problems due to the knee/hip problems, n (%)		
Yes	7811 (76.46)	22,098 (78)
No	2405 (23.54)	6222 (21.96)
Missing	—	11 (0.04)
Knee pain at least every day?, n (%)		
Yes	8213 (80.39)	22,958 (81.03)
No	2003 (19.61)	5373 (18.97)
Missing	—	0 (0)
Afraid that your joints will be damaged from physical activity and exercise?, n (%)		
Yes	1528 (14.96)	4974 (17.56)
No	8688 (85.04)	23,351 (82.42)
Missing	—	6 (0.02)
Pain intensity in the index joint during the last month (VAS^e^ scale 0‐100, no pain to worst pain)		
Mean (SD)	47.19 (21.69)	47.75 (22.49)
Range (min-max)	0.0‐100.0	0.0‐100.0
Missing, n (%)	—	26 (0.09)
Number of painful body areas (collected via pain drawing)		
Mean (SD)	3.79 (3.25)	4.29 (3.83)
Range (min-max)	0.0‐40.0	0.0‐56.0
Missing, n (%)	—	7791 (27.50)
Pain in the lower back (collected via pain drawing), n (%)		
Yes	2686 (26.29)	6105 (21.55)
No	7530 (73.71)	14,435 (50.95)
Missing	—	7791 (27.50)
Are your hip or knee problems so severe that you would like an operation? n (%)		
Yes	1111 (10.88)	4210 (14.86)
No	9105 (89.12)	24,092 (85.04)
Missing	—	29 (0.10)
Working status: are you working or studying? n (%)		
Yes	3059 (29.94)	9387 (33.13)
No	7157 (70.06)	18,944 (66.87)
Missing	—	0 (0)
Sick leave during the last year because of knee or hip problems, n (%)		
Yes	147 (11.23)	3560 (12.57)
No	9069 (88.77)	24,734 (87.30)
Missing	—	37 (0.13)
Have you had a joint replacement in any hip or knee? n (%)		
Yes	939 (9.19)	2251 (7.95)
No	9277 (90.81)	23,768 (83.89)
Missing	—	2312 (8.16)
Frequency of exercise to the point of breathlessness or sweating at least 2‐3 times a week? n (%)		
Yes	5188 (50.78)	14,226 (50.21)
No	5028 (49.22)	14,105 (49.79)
Missing	—	0 (0)
UCLA^f^-physical activity score (from 0 to 10, worst to best)		
Mean (SD)	5.77 (1.78)	5.48 (1.84)
Range (min-max)	1.0‐10.0	1.0‐10.0
Missing, n (%)	—	21 (0.07)
KOOS-12^g^ Quality of life subscale score		
Mean (SD)	46.06 (15.0418)	44.99 (15.5436)
Range (min-max)	0.0‐100.0	0.0‐100.0
Missing, n (%)	—	8 (0.02)
EQ-5D-5L score (–0.757 to 1, worst to best)		
Mean (SD)	0.78 (0.1816)	0.76 (0.2018)
Range (min-max)	–0.38‐1.0	–0.63‐1.0
Missing, n (%)	—	19 (0.06)
General health evaluated via EQ VAS (from 0 to 100, worst to best)		
Mean (SD)	70.37 (18.68)	68.53 (19.29)
Range (min-max)	0.0‐100.0	0.0‐100.0
Missing, n (%)	—	12 (0.04)
Outcome variable		
VAS pain change score from baseline to 3 month follow-up score 100 to 100 from pain got worse to pain got better, 0=no change in pain		
Mean (SD)	14.41 (22.6758)	11.67 (23.44)
Range (min-max)	–87.0‐99.0	–98.0‐100.0
Missing, n (%)	—	10,976 (38.74)

a Not applicable.

b OA: osteoarthritis.

c NSAID: nonsteroidal antiinflammatory drug.

d Yes: Living alone; No: Living with others (partner, family, friends, or others).

e VAS: visual analog scale.

f UCLA: University of California, Los Angeles.

g KOOS-12: Knee Injury and Osteoarthritis Outcome Score.

### Model Development and Model Evaluation

In total, we built 3 models each including a different set of variables (Table 2). The first includes all 34 variables (full model). The second one includes the 11 most important variables identified with the Gini impurity and elbow method (continuous model), highlighting the importance of these baseline variables in making predictions. The third includes the 6 predictive variables, which were chosen based on their importance and clinical reasoning—it is assumed that answering the underlying 11 questions would be applicable in routine clinical practice. These 6 variables are baseline pain, duration of symptoms, the EQ-5D score, time to complete a 40-meter walking test, age, and BMI. Each of those 6 variables contains 1 item but BMI and EQ-5D include 2 and 5 items, respectively.

The model performances are presented in Table 3 based on the average performance on the test sets in a 10-fold cross-validation process. Accordingly, the full model’s average RMSE was 18.56 (SD 0.59), while the coefficient of determination (*R*^2^) was 0.32. The continuous model displayed an RMSE of 18.64 (SD 0.58) and an *R*^2^ of 0.32. Moreover, when considering variables in the concise model, the RMSE and *R*^2^ were 18.76 (SD 0.64) and 0.31, respectively.

**Figure 4.**
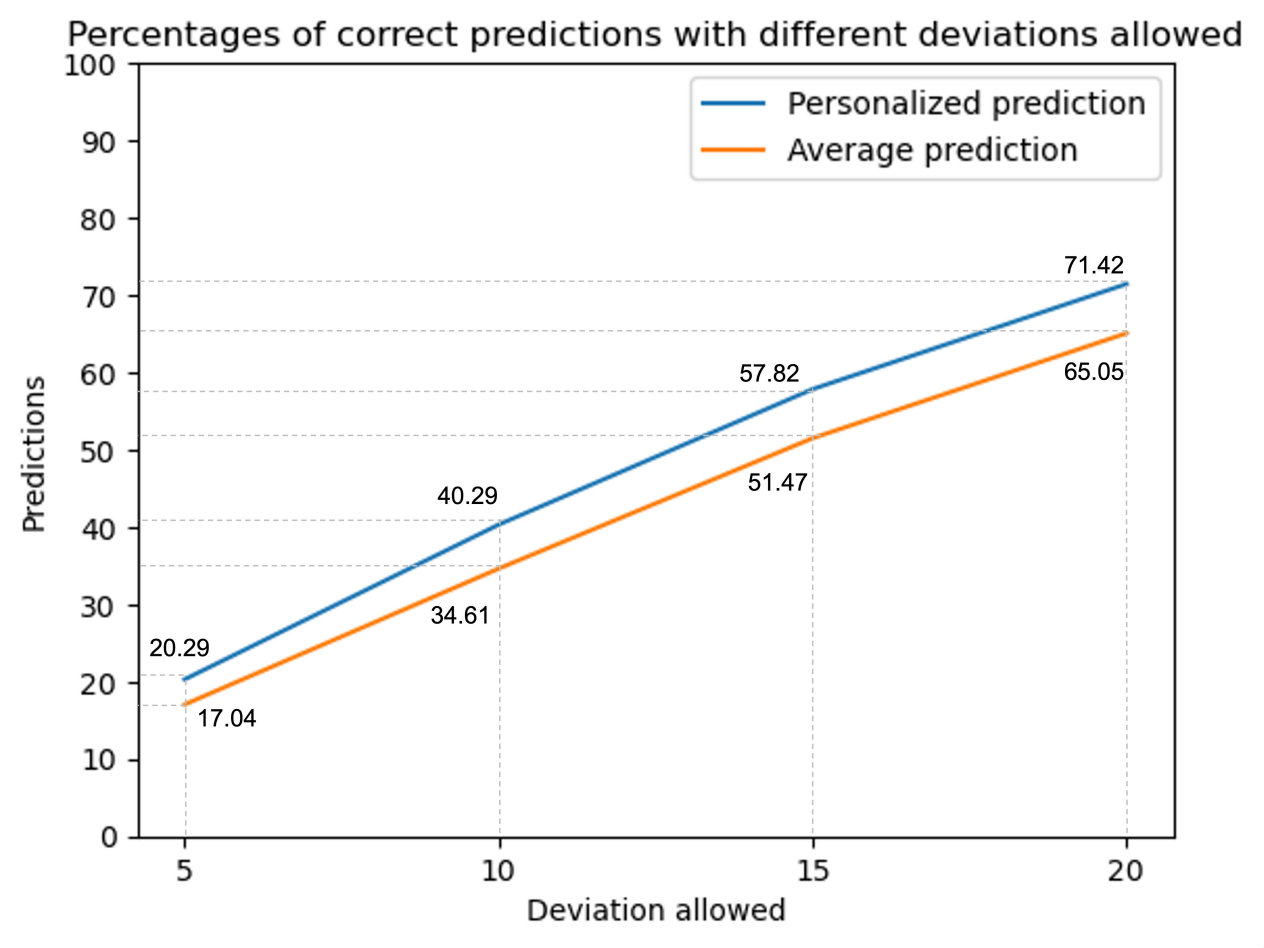
Comparison of the percentage of correct predictions of our concise model and using the average model, allowing deviations between 5 and 20 points.

The comparison between true predictions based on our concise model including the top six most important variables and predictions using the average model is illustrated in Figure 4. It shows a small but distinct advantage of our personalized predictions over average-based predictions. Our personalized prediction method, based on random forest regression, performed better than the average prediction model by a margin of about 7% when allowing a difference of 15 points in each direction between the predicted and the true change on the test data [35]. Specifically, our results showed that the average model predicts 51.47% and our personalized model predicts about 57.82% of the cases correctly. We found that personalized predictions were slightly and consistently better than those based on the average improvement, regardless of the deviation allowed (5 points or 20 points). Table 3 shows correct personalized predictions and correct average predictions within the interval of ±15 points for all models. An illustration using these numerical values is as follows: if a patient, for instance, has a pain score of 45 before GLA:D and our personalized model estimates a change of 20 points, this means that it is estimated that the patient will have pain between 10 and 40 after the program with a 58% certainty.

**Table 2.** Overview of included variables per model.

Name of variable combination	Included variables
Full model	All variables
Continues model	The 11 most important variables were selected by Gini. Identified by variable selection
Concise model for clinical practice	Age, BMI, change in pain, duration of symptoms, time to complete 40 m walking test, EQ-5D score

**Table 3.** Overview of the performance of all random forest regression models.

Variable combination	VAS^a^ pain: GLA:D personalized prediction			
	RMSE^b^	*R* ^2^	Correct personalized predictions (within the interval of ±15 points)	Correct average predictions (within the interval of ±15 points)
Full model	18.56	0.32	58.17%	51.47%
Continues model	18.64	0.32	58.09%	51.47%
Concise model for clinical practice	18.76	0.31	57.82%	51.47%

a VAS: visual analog scale.

b RMSE: root-mean-squared error.

### Supplementary Analyses

Including the 12 additional variables reduced our sample with complete cases to n=1458, since these variables were first added later. However, the evaluation of the variable importance followed a similar pattern highlighting that the now 14 continuous variables were the most important (Figure S2 in Multimedia Appendix 1 [37]). Similarly, the model performances and evaluations remained comparable among all three models (Table 4).

**Table 4.** Overview of performance summary of all random forest regression models of our supplementary analyses, incorporating 46 variables with 12 additional variables relative to the prior model^a^.

Variable combination	VAS^b^ pain: GLA:D personalized prediction			
	RMSE^c^	*R* ^2^	Correct personalized predictions (within the interval of ±15 points)	Correct average predictions (within the interval of ±15 points)
Full model	19.24	0.32	56.37%	49.45%
Continues model	19.25	0.32	56.64%	49.45%
Concise model for clinical practice	19.55	0.30	54.79%	49.45%

a This includes 30 binary variables, 2 categorical variables, and 13 continuous variables.

b VAS: visual analog scale.

c RMSE: root-mean-squared error.

## Discussion

### Principal Findings

In this study, we developed personalized prediction models for changes in knee OA pain after supervised patient education and exercise therapy (GLA:D) and compared them to existing prediction and average-based models. Our findings validate the two previously developed models from Baumbach et al [20] and introduce a new concise personalized prediction model for estimating changes in knee pain intensity among patients with knee OA considering participating in the GLA:D program. Neither the increase in sample size nor the inclusion of additional variables improved the previous prediction model. Nonetheless, our concise model correctly predicted 58% of the cases and demonstrated a 7% improvement in the rate of correct predictions over the use of currently used average values for informing patients about their expected changes in knee pain.

In clinical decision-making, predictive models serve as pivotal tools across various disciplines. These models, akin to those used in cancer prognosis or the optimization of exercise regimens, underline the significance of personalized predictions in enhancing patient outcomes [38-42]. This study delves into this paradigm by comparing prediction models and validating the findings of an earlier study. As in the previous study [20], we used the GLA:D data and presented a full and continuous model to predict personalized outcomes. The latter was chosen based on the variable importance. While we used the Gini impurity and elbow method, the previous study determined the most important variables by the reduction in RMSE for out-of-bag cases and applying the elbow method [20]. Despite the difference in the used method, our findings that the continuous variables were most important align with the previous study. Our outcomes match those of the previous study, despite our model’s sample size being doubled and the variables being reduced by 17 and 4 in the full and continuous model, respectively. For the full and continuous models, there was no difference, and a difference of 0.01 in the *R*^2^ values, respectively. In addition, the RMSE aligned closely. In our supplementary analysis, we incorporated 12 new variables, included in the GLA:D registry between 2017 and 2018, which reduced our sample size to 1458 but maintained consistent variable importance and model performance. Thus, we found that neither an increase in sample size nor the added variables could improve the personalized outcome predictions compared to the earlier study [20].

Recent research [43,44] highlights that patients using analgesics, experiencing constant pain, or preferring the GLA:D program over saline injection demonstrated greater benefits from the program. Unfortunately, our data did not include “constant pain” or “preference for the GLA:D program.” However, we considered “intake of analgesics,” although it was not among the top predictive variables. The random forest method inherently models nonlinear relationships and interactions, enabling relevant subgroup distinctions within the data. This approach avoids reducing sample sizes and maintains robustness by incorporating all relevant variables in the model [45]. Including the variables “constant pain” and “preference for the GLA:D program” in future analyses could provide deeper insights into personalized outcomes and patient stratification. Additionally, it is important to note that our sample probably differs from the randomized controlled trial–based group in the study by Henriksen et al [43,44], as not all patients in our real-world cohort would have agreed to participate in a controlled trial.

To compare the personalized predictions with predictions based on average values, we again used a different approach than the previous prediction study. The previous study took the absolute mean difference and concluded that the difference between their model and the average improvements was not clinically relevant. In this study, we focused on the number of correct predictions, assessing a range of clinical relevance thresholds to determine whether our personalized model surpasses the average model’s performance, despite changing the clinical relevance threshold. Here, we emphasize this incremental progress of increasing the clinical relevance threshold. As a result, allowing a higher deviation from the true change resulted in a higher number of correct predictions, for both our personalized predictions and the average prediction. Overall, we found that our personalized prediction model was able to predict about 7% more cases correctly compared to using average predictions, independent of the threshold allowed from the true change.

However, the question of whether these findings are sufficient for implementation into clinical practice remains open. Existing shared decision-making tools often concentrate on comparing different treatments and assisting patients in making informed choices [18]. Specifically, in the context of OA, where education and exercises are universally recommended and surgery is considered a last resort after the failure of previous treatments, the potential of improved prediction models to alter this recommendation landscape is significant [46]. If future models could reliably predict the likelihood of initial treatment failures for specific patient groups, clinical guidelines to treat OA could evolve. Future models could benefit from including more comprehensive patient characteristics, such as constant pain and treatment preferences [43,44], to further explore patient-specific responses to the GLA:D program. Yet, it is crucial to acknowledge that the current performance of our models is too preliminary for such decisive changes in clinical practice. This underscores the necessity for further research to enhance model accuracy and reliability, as well as to understand the needs and preferences of patients and clinicians, potentially reshaping treatment protocols for OA based on predictive insights.

### Limitations

This study also has some limitations. Selection bias is possible in any registry-based study due to the cases lost to follow-up. However, we only excluded 28.44% due to missing information on the outcome at follow-up, suggesting no serious threats to its external validity [47]. Furthermore, from previous studies using the GLA:D data, we know that these patients do not differ to a clinically relevant level from the included patients [20]. Two periods of technical flaws in data collection were identified, and these missing values, unrelated to patient characteristics or variables, meet the criteria for missing completely at random. Participation in the GLA:D program may also be influenced by factors contributing to selection bias, such as limited accessibility, financial constraints, awareness and referral issues, perceived severity of OA, and language barriers [22,25,48-52]. Our sample’s greater representation of female participants reflects known trends of greater knee OA prevalence and greater participation in exercise programs like GLA:D [53-55]. While this enhances relevance to real-world treatment populations, it should be considered when generalizing findings. This study suggests that the increased sample size does not significantly affect the predictive performance outcome [20]. However, it should be noted that we included fewer variables in comparison to the previous study [20]. These variables were deemed of less relevance and therefore excluded from the registry, nonetheless, they contributed to the predictions of the previous models. Therefore, to be precise, we can only conclude that the increased sample size was able to compensate for the reduced number of variables. Hence, to improve our models’ predictability, new variables would probably need to be integrated. The newly integrated variables (12 additional variables) in our supplementary analyses, led to a reduction in the sample size (n=1458), however, the model performance remained similar. Consequently, we can (only) conclude that the newly added variables could compensate for the reduced sample size.

Based on these observations, we can point toward the limitation of available predictor variables. Implementing more detailed questionnaire-based measures for anxiety and depression might enhance pain predictions [56]. Weight loss is another recommended first-line treatment for individuals with overweight or obesity with knee OA [57] that is known to moderate systemic inflammation [58], extending the questionnaire to enquire about patients’ dietary habits might thus be another possibility to increase the predictive value [59]. However, due to the clinical applicability, the extent of the GLA:D registry questionnaire needs to balance the collection of information against patient burden, adding additional variables with minor importance is not desired. In conclusion, we currently do not know which variables could have an additional major positive influence on the personalized outcome predictions over those already included [60].

### Future Directions and Considerations

The precision of our model aligns with existing clinical studies, suggesting a need for future investigations into the practical value of such predictive accuracy within clinical settings [61]. Current initiatives like the A.S.K. report and Movement is Life have begun to explore the integration of predictive models and patient-reported outcomes to guide shared decision-making, demonstrating the utility of these tools in clinical practice [18,62]. However, there is a noticeable gap in the literature concerning the specific performance benchmarks required for predictive models in physiotherapy to be deemed clinically relevant, particularly in the management of OA. Therefore, before a decision on implementing our model as a web tool into clinical practice can be drawn, guidance on which metrics and predictive values need to be achieved in a model to be feasible for clinical practice needs to be investigated. Furthermore, it remains uncertain if the implementation of a model, like ours, which provides more correct predictions than what is currently used, but still several wrong predictions, is valuable enough for patients and clinicians to be implemented in clinical practice. Further, to the best of our knowledge, it is yet unknown if personalized outcome predictions regarding a treatment lead to different expectations than average outcome predictions in patients. Moreover, on ethical aspects, the consequences of wrong predictions in general on a patient’s treatment, and mental and emotional well-being should be investigated. For patients with estimated worsening pain predicting other health outcomes additionally could be essential to motivate them to participate in the program. For example, prediction tools within GLA:D for secondary outcomes such as physical activity, which changes independently of changes in pain, could support participation rates, given the overarching health benefits associated with increased physical activity [27,60]. Similarly, patients might be more motivated if they knew about the expected changes in physical function and quality of life. Finally, any prediction tool needs to be comprehensively evaluated based on its role in treatment decisions, patient satisfaction, and overall treatment outcomes in clinical settings, before implementation.

### Generalizability

The methodology used in this study, which leverages machine learning techniques to analyze questionnaire data and functional test data for predicting treatment outcomes has been used before and is a promising advancement in the field of personalized medicine [63,64]. This approach is characterized by the selection of predictive variables from data and can be used in similar questioning. Thus, our method extends beyond the specific context of the GLA:D program and could be applied to other exercise therapy programs targeting knee OA, and potentially, for a broader range of conditions [62,65-67].

Generalizing our findings, the model predictions of the GLA:D program for knee OA to other exercise and physiotherapy interventions are challenging. The unique combination of group-based exercises and education in GLA:D may be crucial and not present in all physiotherapeutic settings. Therefore, our outcome predictions specific to GLA:D cannot be expected universally. However, if the core principle of structured exercise therapy and education are incorporated into the treatment of a patient with OA, our model might be applicable. Nonetheless, the personalized predictions would likely deviate even further from the true changes than in patients participating at GLA:D, therefore, they should not be generalized.

### Conclusions

We developed and validated a personalized prediction model for pain intensity changes in patients with knee OA participating in the GLA:D program. Our model’s 58% accuracy is 7% better than using average pain improvement as a prediction. This likely represents the best possible prediction given current knowledge and available data. Although performance is improved, further guidance is needed to determine when such models are suitable for clinical implementation. This study highlights both the potential and current limitations of personalized prediction in knee OA management.

## Supplementary material

10.2196/60162 Multimedia Appendix 1 Additional figures.

## Abbreviations

GLA:D: Good Life with osteoArthritis in Denmark
OA: osteoarthritis
RMSE: root-mean-squared error
TRIPOD: Transparent Reporting of a Multivariable Prediction Model for Individual Prognosis or Diagnosis
VAS: visual analog scale
