# Supplementary material for: Personalized Predictions for Changes in Knee Pain Among Patients With Osteoarthritis Participating in Supervised Exercise and Education: Prognostic Model Study
Source: JMIR Rehabil Assist Technol. 2025 Mar 21;12:e60162. doi: 10.2196/60162 (PMC11951821; doi:10.2196/60162)
Supplement: Multimedia Appendix 1 [file rehab-v12-e60162-s001.docx]

Additional information about this study can be found at [37].

Figures S1 and S2 illustrate the top variables affecting changes in VAS pain scores, ranked by a random forest regressor using Gini impurity.

Figure S1, shows the top variables from a 34-variable model, with the 11 most important variables highlighted and Figure S2, extends the analysis to a 46-variable model, identifying the 14 most important variables.


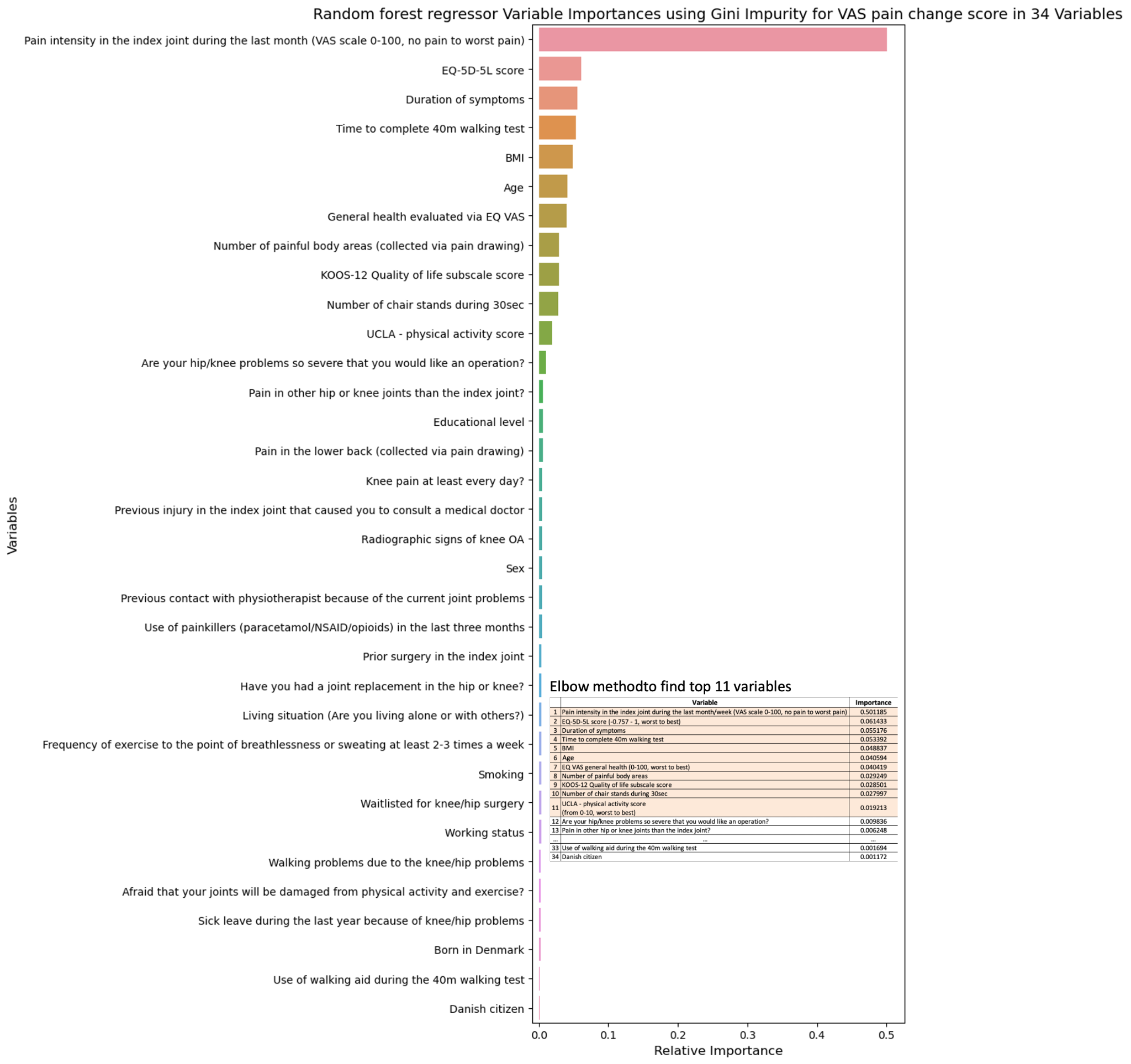


Figure S1: Visual breakdown of the top variables affecting VAS pain change scores, ranked by a random forest regressor using Gini Impurity from a 34 variable, with an inset highlighting the 11 most important variables as determined by the elbow method.

**
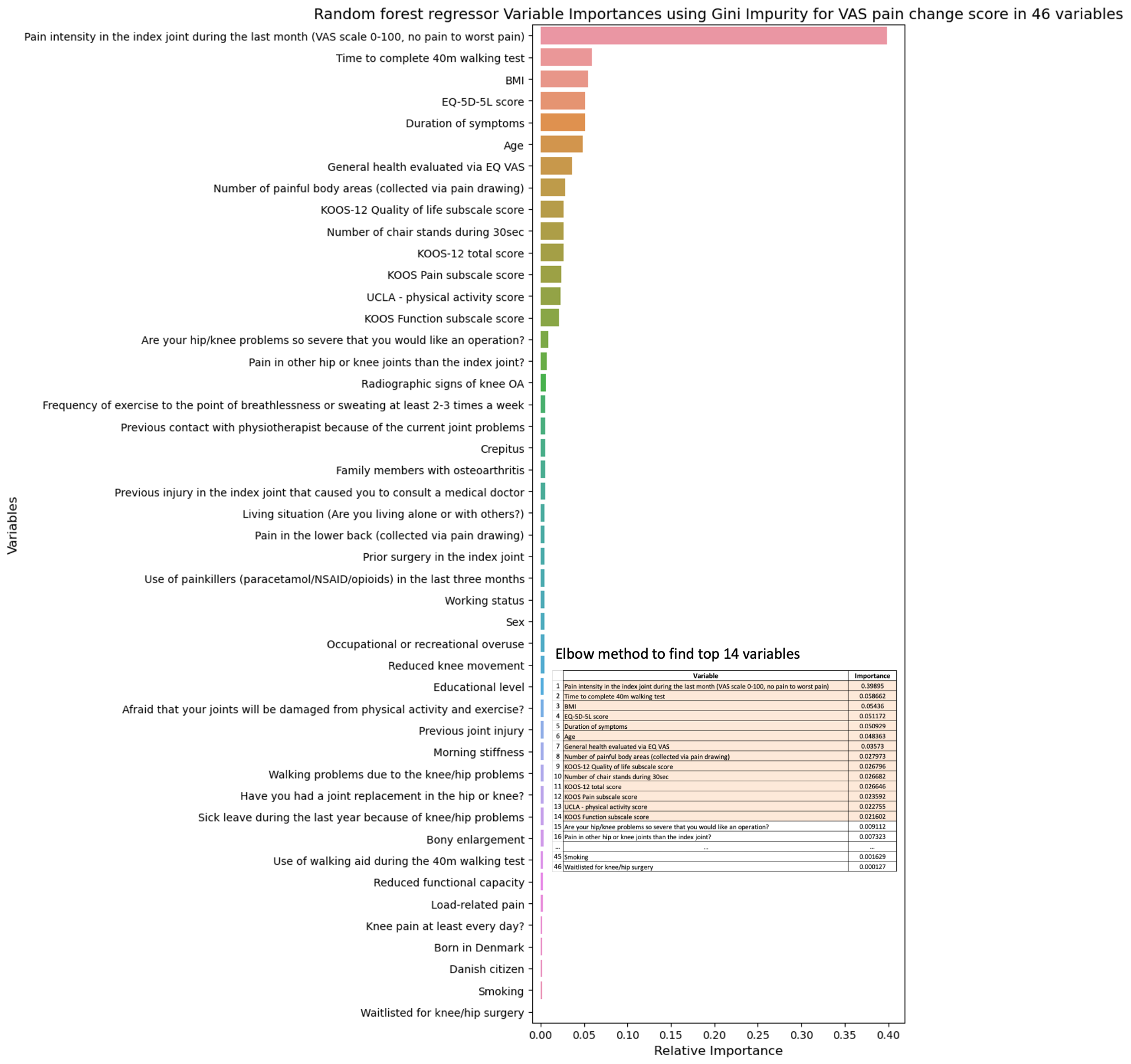
**

Figure S2: Visual breakdown of the top variables affecting VAS pain change scores, ranked by a random forest regressor using Gini Impurity from a 46-variable, with an inset highlighting the 14 most important variables as determined by the elbow method.
